# D-4F, an apolipoprotein A-I mimetic, suppresses IL-4 induced macrophage alternative activation and pro-fibrotic TGF-β1 expression

**DOI:** 10.1080/13880209.2019.1640747

**Published:** 2019-07-23

**Authors:** Xuejiao Song, Ying Shi, Jia You, Zhengshu Wang, Linshen Xie, Chaoxiong Zhang, Jingyuan Xiong

**Affiliations:** a West China School of Public Health and Healthy Food Evaluation Center, Sichuan University, Chengdu, China;; b Research Center for Occupational Respiratory Diseases, West China Fourth Hospital, Sichuan University, Chengdu, China

**Keywords:** Apo A-I mimetic, THP-1, pulmonary fibrosis, monocyte-derived macrophages, M2 macrophage

## Abstract

**Context:** We reported that D-4F, an apolipoprotein A-I (Apo A-I) mimetic polypeptide with 18 d-amino acids, suppressed IL-4 induced macrophage alternative activation and TGF-β1 expression in phorbol 12-myristate 13-acetate (PMA) treated human acute monocytic leukemia cells (THP-1).

**Objective:** Macrophage alternative activation, TGF-β1 and epithelial-mesenchymal transition (EMT) are intensively involved in pulmonary fibrosis. Recent studies demonstrated that Apo A-I resolved established pulmonary fibrotic nodules, and D-4F inhibited TGF-β1 induced EMT in alveolar cells. Therefore, this study evaluated the effects of D-4F on IL-4 induced macrophage alternative activation and TGF-β1 expression.

**Materials and methods:** THP-1 cells were simulated with PMA (100 ng/mL) for 48 h and treated with medium control, IL-4 (20 ng/mL) alone, or IL-4 (20 ng/mL) in the presence of D-4F (1, 5, and 10 μg/mL) for 24 and 48 h. Flow cytometry, RT-PCR and ELISA evaluations were performed to investigate the subsequent effects of D-4F.

**Results:** Compared to stimulation with IL-4 alone, 1, 5, and 10 μg/mL of D-4F reduced alternative activation by 45.38%, 59.98%, and 60.10%, increased TNF-α mRNA levels by 8%, 11%, and 16% and decreased TGF-β1 mRNA levels by 21%, 37%, and 39%, respectively (all *p* ≤ 0.05). In addition, TNF-α protein levels increased from 388 pg/mL (IL-4 alone) to 429, 475, and 487 pg/mL (1, 5, and 10 μg/mL D-4F), while TGF-β1 protein levels dropped from 27.01 pg/mL (IL-4 alone) to 19.15, 12.27, and 10.47 pg/mL (1, 5, and 10 μg/mL D-4F).

**Conclusion:** D-4F suppressed IL-4 induced macrophage alternative activation and pro-fibrotic TGF-β1 expression.

## Introduction

Macrophages are classified into two major phenotypes based on physiological functions and activation mechanisms (Gordon 2003). The M1 phenotype undergoes classical activation through T_h_1 cell cytokines and produces proinflammatory tumour necrosis factor-α (TNF-α) in host defence and anticancer activities. The M2 phenotype is alternatively activated by T_h_2 cell cytokines including interleukin 4 (IL-4) and plays vital roles in promoting cell growth, collagen formation, tissue repairing, and fibrogenesis (Shearer et al. 1997; Song et al. 2000; Wynn and Barron 2010; Wynn and Ramalingam 2012). Upregulation of IL-4 and induction of alveolar M2 macrophage were reported in the lung and bronchoalveolar lavage (BAL) fluid from patients with idiopathic pulmonary fibrosis (IPF), supporting that M2 macrophage is a key mediator controlling fibrogenesis and inhibition of macrophage alternative activation is one of the ideal strategies to treat pulmonary fibrosis (Prasse et al. 2006; Pechkovsky et al. 2010). The irreversible pulmonary fibrosis is destructive and often leads to progressive dyspnea and gradual deterioration in lung function (Gross and Hunninghake 2001; Meltzer and Noble 2008). Although several anti-fibrotic agents were reported to partially relieve symptoms and detain fibrosis progression, currently no pharmaceutical is clinically available to resolve established fibrotic nodules and completely cure fibrosis (Iyer et al. 1998; Demedts et al. 2005; Choe et al. 2010; Pini et al. 2010).

A breakthrough was revealed in a high-throughput proteomic study, in which apolipoprotein A-I (Apo A-I) was significantly diminished in BAL fluid from IPF patients (Kim et al. 2010). Intranasal replenishing of Apo A-I was shown to ameliorate bleomycin induced lung injury in mice. More importantly, continuing investigation using transgenic mice demonstrated for the first time that silica-induced alveolar fibrotic nodules were partially resolved by Apo A-I local overexpression (Lee et al. 2013). However, despite that Apo A-I holds exceptional anti-fibrotic potential, genetic therapy for human is ethically challenging and far from readily available. Therefore, effective and practical Apo A-I substitutes are in demand to fight against pulmonary fibrosis.

D-4F, a synthetic Apo A-I mimetic with 18 d-amino acids, was claimed safe in human clinical trials for coronary heart disease (Bloedon et al. 2008). Since Apo A-I is the major component of plasma high-density lipoprotein (HDL), D-4F was mainly investigated in treating atherosclerosis and cardiovascular disorders (Navab et al. 2005, 2006; Getz and Reardon 2011). To date, only a few studies have shed light on the effects of D-4F on respiratory diseases. In the asthmatic model, D-4F was shown to decrease airway hyper-responsiveness and oxidative stress (Nandedkar et al. 2011). In alveolar cells, D-4F was reported to inhibit transforming growth factor β1 (TGF-β1) induced epithelial-mesenchymal transition (EMT) (You et al. 2016). As EMT is extensively documented in the pathogenesis of pulmonary fibrosis, D-4F was speculated to exert anti-fibrotic effect similar to Apo A-I (Selman and Pardo 2003; Kasai et al. 2005; Willis et al. 2005). Given M2 macrophage and TGF-β1 are the key regulators of fibrosis and ubiquitously up-regulated in IPF patients, whether D-4F can interfere with macrophage activation and TGF-β1 expression is of particular interest.

Datta and colleagues reported that Apo A-I and mimetic 4F attenuate lipopolysaccharide (LPS)-induced inflammation by initiating functional changes in monocyte-derived macrophages (MDMs), thereby improve HDL function and inhibit atherosclerotic lesion (Navab et al. 2002; Smythies et al. 2010; White et al. 2012). Nevertheless, the milieu and timing of inflammatory events in pulmonary fibrosis are vastly different from cardiovascular diseases (Wilson and Wynn 2009). In addition, D-4F exerts superior pharmacokinetics and biosafety than its 4F counterparts due to the lack of peptidase for d-amino acids in mammalian system (Bloedon et al. 2008). To date, no study has demonstrated any observation between D-4F and fibrosis related M2 macrophages. Thus, this work was aimed to preliminarily explore the effects of D-4F on IL-4 induced macrophage alternative activation and pro-fibrotic TGF-β1 expressions, in order to shed light on the basis for future possible treatment of pulmonary fibrosis.

## Materials and methods

### Chemicals and reagents

Recombinant human IL-4, human TNF-α and TGF-β1 ELISA kits were purchased from R&D Systems (Minneapolis, MN). F4/80 antibody (BM8) [FITC] and CD163 antibody (6E10.1G6) [Alexa Fluor (R) 647] were purchased from Novus Biologicals (Minneapolis, MN, USA). PrimeScript cDNA synthesis kit and Premix Ex taq kit were from Takara (Dalian, China). D-4F (Ac-DWFKAFYDKVAEKFKEAF-NH_2_) was synthesized by Scilight-Peptide (Beijing, China).

### Cell lines and culture

The human acute monocytic leukemia cell line, THP-1 (The American Type Culture Collection, Manassas, VA, USA), was maintained in RPMI-1640 medium containing 10% FBS, 100 U/mL penicillin and 100 μg/mL streptomycin in a humidified 5% CO_2_ atmosphere at 37 °C. When 70–80% confluence was reached, cell passages were conducted. All experiments were performed using cells after three or four passages.

### Alternatively activated macrophages and stimulations

Subcultures of THP-1 cells were seeded in six-well culture dishes at a cell density of 5 × 10^4^ per dish for subsequently stimulation and analysis. THP-1 cells were treated with 100 ng/mL of PMA for 48 h and stimulated with 20 ng/mL of IL-4. To investigate the effect of D-4F on M2 activation, cells were treated for 24 and 48 h with control, IL-4 (20 ng/mL) alone, or IL-4 (20 ng/mL) in the presence of D-4F (1, 5, and 10 μg/mL).

### Flow cytometric analysis

Under different stimulation conditions mentioned above, cells (1 × 10^6^) were incubated with antibodies or control in dark for 30 min at 4 °C. Then, the cells were washed with PBS buffer twice, fixed with 1% paraformaldehyde, and subjected to flow cytometric analysis. The data were analysed by FlowJo software (Tree Star, Ashland, OR, USA).

### RT-PCR and transcriptional analysis

Primer pairs in the RT-PCR were (5′ACCAACTATTGCTTCAGCTC) and (5′CTTGCAGGAGCGCACGATCA) for TGF-β1, (5′ATCTACCTGGGAGGCGTCTT) and (5′GAGTGGCACAAGGAACTGGT) for TNF-α, (5′GGACTTCGAGCAAGAGATGG) and (5′AGCACTGTGTTGGCGTACAG) for β-actin. β-Actin was used as the internal control. Arithmetic formula 2^–ΔΔCT^ was used to quantify the relative changes in the transcription of TNF-α and TGF-β1.

### ELISA and translational analysis

Under varying conditions mentioned above, cells were harvested and centrifuged at 1000 g for 10 min, followed by ELISA analysis on collected supernatant according to the manufacturer’s instruction.

### Statistical analysis

The collected data were reported as mean ± standard error of mean (SEM). Experiments were conducted in triplicates for all the quantifications. Differences among groups were analysed using one-way ANOVA test. Statistical analyses between two groups were conducted using Dunnet *t*-test. Significance was assumed when *p* ≤ 0.05.

## Results

### IL-4 induces alternative activation of PMA treated THP-1 cells

**Figure 1.**
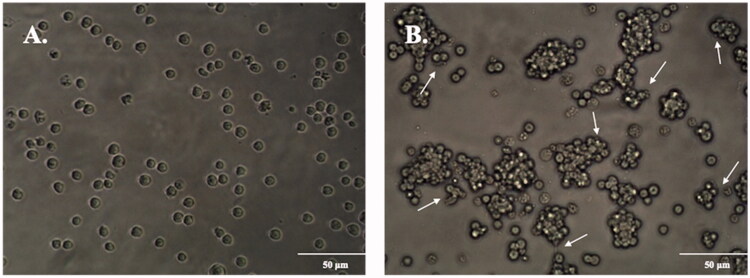
PMA induces the transition from THP-1 cells to MDMs. (A) Control cells treated with culture medium alone demonstrated a globular appearance with no cell-cell interactions. (B) Cells treated with 100 ng/mL of PMA assumed adherent morphology with apparent cell aggregation and pseudopodia (marked by arrows), representing MDMs.

**Figure 2.**
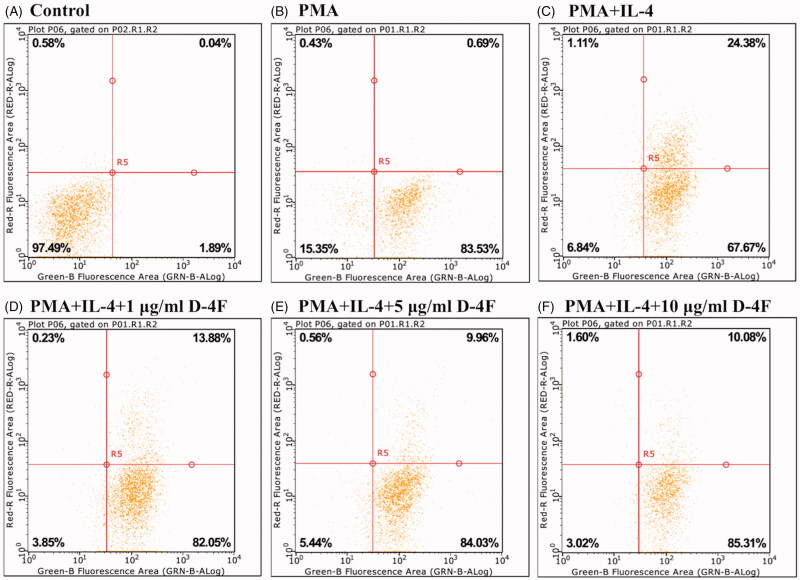
IL-4 alternatively activates MDMs and D-4F suppresses IL-4 induced alternative activation. Flow cytometry was performed on cells treated with medium control (A), PMA (B), IL-4 (C), IL-4 and 1 μg/mL of D-4F (D), IL-4 and 5 μg/mL of D-4F (E), and IL-4 and 10 μg/mL of D-4F (F). Green-B fluorescence area represented cells labeled with macrophage specific F4/80 antibody (FITC) and Red-R fluorescence area represented cells reacted with M2 macrophage specific CD163 antibody (Alexa Fluor).

In order to analyse the effects of D-4F on M2 macrophages, valid macrophage alternative activation was firstly established and evaluated. THP-1 cells were incubated with 100 ng/mL of PMA for 48 h to obtain MDMs. In the absence of PMA, cells were isolated and suspended with a globular morphology representing typical monocyte (Figure 1(A)). In the presence of PMA, cells were adherent and aggregated with observable pseudopodia resembling MDMs (Figure 1(B)). This transition was confirmed by flow cytometry (Figure 2(A,B)). Under the stimulation of PMA, 83.53% of the cells were identified as MDMs based on their expression of macrophage specific F4/80 antibody. The MDMs were then treated with 20 ng/mL of IL-4 for 48 h to induce alternative activation, followed by flow cytometric analysis (Figure 2(C)). In the presence of IL-4, 92.05% of the cells were characterized as MDMs, 67.67% of cells were MDMs without expression of M2 phenotypic surface marker CD163 and 24.38% of the cells were identified as M2 macrophages, leading to a 26.49% alternative activation. These results confirmed that IL-4 was able to induce alternative activation in MDMs.

### D-4F suppresses IL-4 induced alternative activation in PMA treated THP-1 cells

Flow cytometric analysis was performed on MDMs in the presence of IL-4 (20 ng/mL) and varying concentrations of D-4F (1, 5, and 10 μg/mL), in order to investigate the effects of D-4F on IL-4 induced macrophage alternative activation. Under the stimulation of IL-4 and 1 μg/mL of D-4F, 95.93% of the cells were identified as MDMs, 82.05% of cells were non-M2 MDMs, and 13.88% of the cells showed M2 features, resulting in a 14.47% alternative activation (Figure 2(D)). In the presence of IL-4 and 5 μg/mL of D-4F, 93.99% of the cells were MDMs, 84.03% of cells were MDMs without CD163 expression, and 9.96% of the cells transformed into M2 macrophages, causing a 10.60% alternative activation (Figure 2(E)). When IL-4 and 10 μg/mL of D-4F were present, 95.39% of the cells were MDMs, 82.31% of cells were MDMs in absence of M2 characteristics, and 10.08% of the cells were M2 macrophages, leading to a 10.57% alternative activation (Figure 2(F)). Compared to the 24.49% alternative activation when MDMs were incubated with IL-4 alone (Figure 2(C)), addition of 1, 5, and 10 μg/mL of D-4F led to a 45.38%, 59.98%, and 60.10% reduction in alternative activation, respectively. These results manifested that D-4F was able to partially suppress IL-4 induced macrophage alternative activation and indicated that the phenotypical inhibitory effects of D-4F might be saturating at a concentration of 5 μg/mL.

### D-4F increases TNF-α transcription and translation in IL-4 induced M2 macrophages

TNF-α is a pro-inflammatory cytokine closely associated with classically activated macrophage. The expression of TNF-α may serve as an indication for the differentiation and function of macrophages. In order to evaluate effects of D-4F on the transcription and translation of TNF-α in IL-4 induced M2 macrophages, RT-PCR and ELISA analysis were performed.

**Figure 3.**
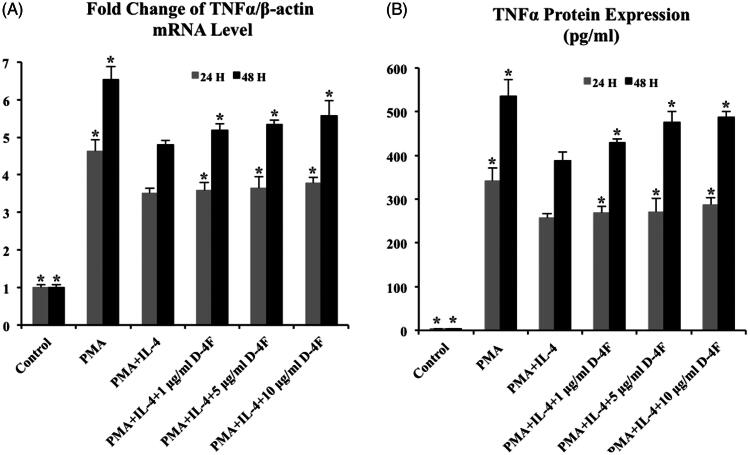
D-4F increases TNF-α transcription and translation in IL-4 induced M2 macrophages. Compared to the cells treated with IL-4 alone, when cells were treated with IL-4 and D-4F (1, 5 and 10 μg/mL), (A) RT-PCR showed that the relative mRNA levels of TNF-α increased 2%, 4% and 8% in 24 h incubation (grey bar) and increased 8%, 11% and 16% in 48 h incubation (black bar) and (B) ELISA analysis demonstrated that the TNF-α protein expression increased 4%, 6% and 12% in 24 h incubation (grey bar) and increased 11%, 22% and 26% in 48 h incubation (black bar). Asterisk indicated significant difference (*p* ≤ 0.05) when comparing to the cells treated with PMA and IL-4 (MDMs with the highest level of alternative activation).

THP-1 cells were treated with PMA for 48 h to induce MDMs, followed by treatment with medium control, IL-4 alone, or IL-4 and varying concentrations of D-4F (1, 5, and 10 μg/mL) for 24 and 48 h. The relative mRNA levels of TNF-α were calculated against β-actin. Apparent down-regulation of TNF-α mRNA was noted in MDMs treated with IL-4 for 24 and 48 h, indicating that the transcription of TNF-α was inhibited in IL-4 induced M2 macrophages (Figure 3(A)). When both D-4F and IL-4 were incubated with MDMs for 24 and 48 h, increases in TNF-α mRNA level were observed with increasing concentrations of D-4F compared to MDMs treated with IL-4 alone. When MDMs were treated with IL-4 and 1, 5, and 10 μg/mL of D-4F for 48 h, the relative TNF-α mRNA levels increased 8%, 11%, and 16%, respectively (Figure 3(A)). These results suggested that the IL-4 induced down-regulation of TNF-α was hindered at transcription level by D-4F.

To investigate TNF-α translation, the supernatants of treated cells were collected and quantified by ELISA analysis. When MDMs were incubated with IL-4 for 24 and 48 h, TNF-α levels decreased to 256 and 388 pg/mL, respectively. The 25% and 27% reduction signified that IL-4 was able to suppress TNF-α expression and pro-inflammatory differentiation of macrophages (Figure 3(B)). When both D-4F and IL-4 were incubated with MDMs for 24 and 48 h, increases of TNF-α expression were more substantial with higher concentrations of D-4F. When MDMs were stimulated with IL-4 and 1, 5, and 10 μg/mL of D-4F for 48 h, the TNF-α concentrations were 429, 475, and 487 pg/mL, demonstrating an increase of 11%, 22%, and 26%, respectively (Figure 3(B)). These results showed that D-4F reversed IL-4 triggered TNF-α down-regulation at translation level and might possess counter effects against IL-4 during macrophage alternative activation.

### D-4F reduces TGF-β1 transcription and translation in IL-4 induced M2 macrophages

TGF-β1 is a pro-fibrotic cytokine strongly related to alternatively activated macrophage. The expression of TGF-β1 not only reflects macrophage activation but it also initiates EMT and affects the progression of pulmonary fibrosis. Therefore, transcriptional and translational studies were conducted to determine whether D-4F has impact on TGF-β1 expression in IL-4 induced M2 macrophages.

**Figure 4.**
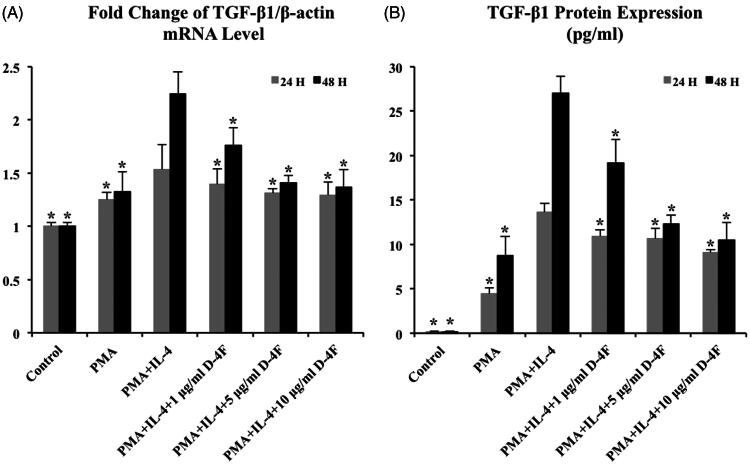
D-4F suppresses TGF-β1 transcription and translation in IL-4 induced M2 macrophages. Compared to the cells treated with IL-4 alone, when cells were treated with IL-4 and D-4F (1, 5 and 10 μg/mL), (A) RT-PCR showed that the relative mRNA levels of TGF-β1 decreased 9%, 15% and 16% in 24 h incubation (grey bar) and decreased 21%, 37% and 39% in 48 h incubation (black bar) and (B) ELISA analysis demonstrated that the TGF-β1 protein expression decreased 20%, 22% and 33% in 24 h incubation (grey bar) and decreased 29%, 55% and 61% in 48 h incubation (black bar). Asterisks indicate significant differences (*p* ≤ 0.05) when comparing to the cells treated with PMA and IL-4 (MDMs with the highest level of alternative activation).

Results from RT-PCR analysis showed that TGF-β1 mRNA levels were significantly increased in IL-4 treated MDMs compared to the control, manifesting that IL-4 was able to facilitate TGF-β1 transcription in MDMs (Figure 4(A)). When cells were treated with both D-4F and IL-4 for 24 h, the relative levels of TGF-β1 mRNA decreased with increasing concentrations of D-4F compared to cells treated with IL-4 alone. When the stimulation of IL-4 and 1, 5, and 10 μg/mL of D-4F were extended to 48 h, the relative TGF-β1 mRNA levels dropped 21, 37, and 39%, respectively (Figure 4(A)). These results indicated that D-4F obstructed the IL-4 induced TGF-β1 transcription in MDMs.

Results from ELISA analysis illustrated that TGF-β1 translation was drastically higher in the presence of IL-4 than that in the control. When MDMs were stimulated by IL-4 for 24 and 48 h, TGF-β1 protein levels rose to 13.62 and 27.01 pg/mL, respectively, supporting the capability of IL-4 to induce the differentiation of pro-fibrotic M2 macrophages (Figure 4(B)). When both D-4F and IL-4 were incubated with MDMs for 24 and 48 h, decrease in TGF-β1 expression were more pronounced with increasing concentrations of D-4F. When MDMs were stimulated with IL-4 and 1, 5, and 10 μg/mL of D-4F for 48 h, the TGF-β1 concentrations were 19.15, 12.27, and 10.47 pg/mL, resulting in a reduction of 29%, 55%, and 61%, respectively (Figure 4(B)). These results suggested that D-4F suppressed TGF-β1 translation in IL-4 induced M2 macrophages, providing feasible anti-fibrotic solution.

## Discussion

This study showed for the first time that D-4F, an Apo A-I mimetic with desirable properties of pharmacokinetics and biosafety, suppressed IL-4 induced macrophage alternative activation and TGF-β1 expression in PMA treated THP-1 cells. PMA was able to induce MDMs in THP-1 cells based on the morphological and flow cytometric characterizations. D-4F was able to prevent the expression of M2 macrophage specific surface marker CD163 on MDMs. In addition, D-4F significantly reversed the IL-4 induced down-regulation of M1 macrophage associated TNF-α and suppressed the up-regulation of pro-fibrotic TGF-β1. When IL-4 treated MDMs were incubated with high concentration of D-4F (10 μg/mL) for 48 h, the mRNA and protein levels of TNF-α increased 16% and 26%, while the mRNA and protein levels of TGF-β1 decreased 39% and 61%. When IL-4 treated MDMs were incubated with low concentration of D-4F (1 μg/mL) for 24 h, the effects were less evident. The reported results in the present study supported that D-4F might possess anti-fibrotic effects by inhibiting macrophage alternative activation and TGF-β1 expression.

D-4F was originally synthesized based on the structure of Apo A-I, aiming to reproduce Apo A-I functions and treat disorders caused by Apo A-I deficiency. Apo A-I is the major component of HDL, exerting anti-inflammatory property that is beneficial to cardiovascular diseases including atherosclerosis (Kwiterovich 1998). Accordingly, research has been intensively focused on the effects of D-4 on cardiovascular disorders. D-4F was shown to bind pro-inflammatory fatty acid hydroperoxides and oxidized phospholipids, improve anti-inflammatory functions of HDL and decrease atherosclerotic lesions (Navab et al. 2005, 2006; Anantharamaiah et al. 2007; Getz and Reardon 2011). The first human clinical trials of D-4F demonstrated that 500 mg of single D-4F administration was safe and well tolerated in patients with high-risk of coronary heart disease (Bloedon et al. 2008). This inspiring work underlies numerous possibilities for clinical applications of D-4F.

Recently, a few pioneering studies switched the focus to the effects of D-4F on respiratory diseases. D-4F was reported to decrease pulmonary inflammation, oxidative stress, and airway hyper responsiveness in experimental asthma induced by ovalbumin sensitization, hinting the application of D-4F on asthma relief (Nandedkar et al. 2011). Furthermore, the expression of TGF-β1 was disrupted by intranasal treatment of D-4F in asthmatic animal model, inferring D-4F might also act on TGF-β1 related disorders. In alveolar epithelial cells, D-4F was speculated to hold promise for the treatment of pulmonary fibrosis due to the inhibition of TGF-β1 induced EMT (You et al. 2016). In human type II pneumocytes, D-4F was shown to reduce the inflammatory responses caused by influenza infection (Van Lenten et al. 2004). Similar anti-inflammatory properties of Apo A-I mimetic 4F were reported in MDMs, validating its vascular protective effects (Smythies et al. 2010). MDMs were treated with relatively low concentration of 4F for 7 d, and transcriptions of 8 phenotypical genes were measured. In 4F treated MDMs, the expression of seven genes was similar to those in M2 macrophages, while the expression of the other gene resembled that in M1 macrophage. These contradicting results indicated that 4F treated MDMs primarily showed M2 phenotype while shared some characteristics of M1 phenotype, leading to the assumption that 4F was able to induce anti-inflammatory differentiation in MDMs (Smythies et al. 2010). However, the characteristics of D-4F cannot be simply assumed to be the same as 4F due to the different three-dimensional structures. The specific effect of D-4F on IL-4 induced macrophage alternative activation has never been addressed. In addition, the pathogenesis and microenvironment of inflammatory cardiovascular disorders is not comparable to pulmonary fibrosis.

In IPF patients, absence of inflammation was observed, and the M2 macrophages were found to be the predominant phenotype, supporting the theory that the timing of inflammatory events and M2 macrophages play determinate roles in pulmonary fibrosis (Selman et al. 2001; Wilson and Wynn 2009). Induction of M2 macrophages contributes to local TGF-β1 expression, which in turn triggers EMT and initiates fibrosis. On the contrary, the presence of phagocytic macrophages assists fibroblast clearance and decreases TGF-β1 expression (Moodley et al. 2003). Therefore, late-stage inflammation could actually be favourable in resolution of dysregulated tissue repairing and fibrosis (Wilson and Wynn 2009). Theoretically, substances that can decrease the amount of M2 macrophages, induce late-stage inflammation and reduce the level of TGF-β1 could potentially be candidates for treatment of pulmonary fibrosis. The present study investigated the effects of D-4F on IL-4 induced macrophage alternative activation. D-4F was reported to directly reduce the amount of IL-4 induced M2 macrophages and TGF-β1 expression, suggesting antagonist effects against IL-4 and anti-fibrotic potential. D-4F was also shown to rectify the IL-4 induced TNF-α down-regulation, which might be beneficial to late-stage inflammation and fibrosis resolvation.

Pulmonary fibrosis is a malignant and multifactorial disorder with poor prognosis, and no clinical treatment can reverse the established fibrosis once formed. Global proteomic analysis on 14 IPF patients reported significant reduction of Apo A-I in BAL fluid (Kim et al. 2010). Analysis on transgenic mice bearing human Apo A-I showed that the volume of fibrotic nodules, TGF-β1 expression and collagen deposition were distinctly reduced when Apo A-I was locally expressed 15 days after silica exposure, suggesting resolution of pulmonary fibrotic nodule (Lee et al. 2013). Although Apo A-I is hitherto the only reported substance that can partially resolve experimental lung fibrotic nodules, obtaining sufficient quantities of Apo A-I is impractical, let alone the poor pharmacokinetic properties. Instead, Apo A-I mimetic D-4F is free from intrinsic enzymatic degradation and clinically safe in human clinical trials. However, in order to be considered as a realistic therapeutic approach for pulmonary fibrosis, further studies on the side effects of D-4F and *in vivo* validations should be performed.

In conclusion, the present study showed that D-4F effectively suppresses IL-4 induced macrophage alternative activation and pro-fibrotic TGF-β1 expression.

## References

[CIT0001] Anantharamaiah GM, Mishra VK, Garber DW, Datta G, Handattu SP, Palgunachari MN, Chaddha M, Navab M, Reddy ST, Segrest JP, et al. 2007. Structural requirements for antioxidative and anti-inflammatory properties of apolipoprotein A-I mimetic peptides. J Lipid Res. 48:1915–1923. 17570869 10.1194/jlr.R700010-JLR200

[CIT0002] Bloedon LT, Dunbar R, Duffy D, Pinell-Salles P, Norris R, DeGroot BJ, Movva R, Navab M, Fogelman AM, Rader DJ. 2008. Safety, pharmacokinetics, and pharmacodynamics of oral apoA-I mimetic peptide D-4F in high-risk cardiovascular patients. J Lipid Res. 49:1344–1352. 18323573 10.1194/jlr.P800003-JLR200 PMC2386905

[CIT0003] Choe JY, Jung HJ, Park KY, Kum YS, Song GG, Hyun DS, Park SH, Kim SK. 2010. Anti-fibrotic effect of thalidomide through inhibiting TGF-beta-induced ERK1/2 pathways in bleomycin-induced lung fibrosis in mice. Inflamm Res. 59:177–188. 19757088 10.1007/s00011-009-0084-9

[CIT0004] Demedts M, Behr J, Buhl R, Costabel U, Dekhuijzen R, Jansen HM, MacNee W, Thomeer M, Wallaert B, Laurent F, et al. 2005. High-dose acetylcysteine in idiopathic pulmonary fibrosis. N Engl J Med. 353:2229–2242. 16306520 10.1056/NEJMoa042976

[CIT0005] Getz GS, Reardon CA. 2011. Apolipoprotein A-I and A-I mimetic peptides: a role in atherosclerosis. J Inflamm Res. 4:83–92. 22096372 10.2147/JIR.S12983 PMC3218745

[CIT0006] Gordon S. 2003. Alternative activation of macrophages. Nat Rev Immunol. 3:23–35. 12511873 10.1038/nri978

[CIT0007] Gross TJ, Hunninghake GW. 2001. Idiopathic pulmonary fibrosis. N Engl J Med. 345:517–525. 11519507 10.1056/NEJMra003200

[CIT0008] Iyer SN, Margoli SB, Hyde DM, Giri SN. 1998. Lung fibrosis is ameliorated by pirfenidone fed in diet after the second dose in a three-dose bleomycin-hamster model. Exp Lung Res. 24:119–132. 9457473 10.3109/01902149809046058

[CIT0009] Kasai H, Allen JT, Mason RM, Kamimura T, Zhang Z. 2005. TGF-beta1 induces human alveolar epithelial to mesenchymal cell transition (EMT). Respir Res. 6:56–71. 15946381 10.1186/1465-9921-6-56 PMC1177991

[CIT0010] Kim TH, Lee YH, Kim KH, Lee SH, Cha JY, Shin EK, Jung S, Jang AS, Park SW, Uh ST, et al. 2010. Role of lung apolipoprotein A-I in idiopathic pulmonary fibrosis: antiinflammatory and antifibrotic effect on experimental lung injury and fibrosis. Am J Respir Crit Care Med. 182:633–642. 20463180 10.1164/rccm.200905-0659OC

[CIT0011] Kwiterovich PO. 1998. The antiatherogenic role of high-density lipoprotein cholesterol. Am J Cardiol. 82:13Q–21Q. 9819099 10.1016/s0002-9149(98)00808-x

[CIT0012] Lee EH, Lee EJ, Kim HJ, Jang A, Koh E, Uh ST, Kim Y, Park SW, Park CS. 2013. Overexpression of apolipoprotein A1 in the lung abrogates fibrosis in experimental silicosis. PLoS ONE. 8:e55827. 23409054 10.1371/journal.pone.0055827 PMC3568133

[CIT0013] Meltzer EB, Noble PW. 2008. Idiopathic pulmonary fibrosis. Orphanet J Rare Dis. 3:8. 18366757 10.1186/1750-1172-3-8 PMC2330030

[CIT0014] Moodley Y, Rigby P, Bundell C, Bunt S, Hayashi H, Misso N, McAnulty R, Laurent G, Scaffidi A, Thompson P, et al. 2003. Macrophage recognition and phagocytosis of apoptotic fibroblasts is critically dependent on fibroblast-derived thrombospondin 1 and CD36. Am J Pathol. 162:771–779. 12598312 10.1016/S0002-9440(10)63874-6 PMC1868087

[CIT0015] Nandedkar SD, Weihrauch D, Xu H, Shi Y, Feroah T, Hutchins W, Rickaby DA, Duzgunes N, Hillery CA, Konduri KS, et al. 2011. D-4F, an apoA-1 mimetic, decreases airway hyperresponsiveness, inflammation, and oxidative stress in a murine model of asthma. J Lipid Res. 52:499–508. 21131532 10.1194/jlr.M012724 PMC3035686

[CIT0016] Navab M, Anantharamaiah GM, Hama S, Garber DW, Chaddha M, Hough G, Lallone R, Fogelman AM. 2002. Oral administration of an Apo A-I mimetic peptide synthesized from D-amino acids dramatically reduces atherosclerosis in mice independent of plasma cholesterol. Circulation. 105:290–292. 11804981 10.1161/hc0302.103711

[CIT0017] Navab M, Anantharamaiah GM, Reddy ST, Hama S, Hough G, Grijalva VR, Yu N, Ansell BJ, Datta G, Garber DW, et al. 2005. Apolipoprotein A-I mimetic peptides. Arterioscler Thromb Vasc Biol. 25:1325–1331. 15831812 10.1161/01.ATV.0000165694.39518.95

[CIT0018] Navab M, Anantharamaiah GM, Reddy ST, Fogelman AM. 2006. Apolipoprotein A-I mimetic peptides and their role in atherosclerosis prevention. Nat Clin Pract Cardiovasc Med. 3:540–547. 16990839 10.1038/ncpcardio0661

[CIT0019] Pechkovsky DV, Prasse A, Kollert F, Engel KM, Dentler J, Luttmann W, Friedrich K, Müller-Quernheim J, Zissel G. 2010. Alternatively activated alveolar macrophages in pulmonary fibrosis-mediator production and intracellular signal transduction. Clin Immunol. 137:89–101. 20674506 10.1016/j.clim.2010.06.017

[CIT0020] Pini A, Shemesh R, Samuel CS, Bathgate RA, Zauberman A, Hermesh C, Wool A, Bani D, Rotman G. 2010. Prevention of bleomycin-induced pulmonary fibrosis by a novel antifibrotic peptide with relaxin-like activity. J Pharmacol Exp Ther. 335:589–599. 20826567 10.1124/jpet.110.170977

[CIT0021] Prasse A, Pechkovsky DV, Toews GB, Jungraithmayr W, Kollert F, Goldmann T, Vollmer E, Müller-Quernheim J, Zissel G. 2006. A vicious circle of alveolar macrophages and fibroblasts perpetuates pulmonary fibrosis via CCL18. Am J Respir Crit Care Med. 173:781–792. 16415274 10.1164/rccm.200509-1518OC

[CIT0022] Selman M, King TE, Pardo A. 2001. Idiopathic pulmonary fibrosis: prevailing and evolving hypotheses about its pathogenesis and implications for therapy. Ann Intern Med. 134:136–151. 11177318 10.7326/0003-4819-134-2-200101160-00015

[CIT0023] Selman M, Pardo A. 2003. The epithelial/fibroblastic pathway in the pathogenesis of idiopathic pulmonary fibrosis. Am J Respir Cell Mol Biol. 29:S93–S97. 14503564

[CIT0024] Shearer JD, Richards JR, Mills CD, Caldwell MD. 1997. Differential regulation of macrophage arginine metabolism: a proposed role in wound healing. Am J Physiol. 272:E181–E190. 9124321 10.1152/ajpendo.1997.272.2.E181

[CIT0025] Smythies LE, White CR, Maheshwari A, Palgunachari MN, Anantharamaiah GM, Chaddha M, Kurundkar AR, Datta G. 2010. Apolipoprotein A-I mimetic 4F alters the function of human monocyte-derived macrophages. Am J Physiol Cell Physiol. 298:C1538–C1548. 20219948 10.1152/ajpcell.00467.2009 PMC2889631

[CIT0026] Song E, Ouyang N, Horbelt M, Antus B, Wang M, Exton MS. 2000. Influence of alternatively and classically activated macrophages on fibrogenic activities of human fibroblasts. Cell Immunol. 204:19–28. 11006014 10.1006/cimm.2000.1687

[CIT0027] Van Lenten BJ, Wagner AC, Navab M, Anantharamaiah GM, Hui EK, Nayak DP, Fogelman AM. 2004. D-4F, an apolipoprotein A-I mimetic peptide, inhibits the inflammatory response induced by influenza A infection of human type II pneumocytes. Circulation. 110:3252–3258. 15533864 10.1161/01.CIR.0000147232.75456.B3

[CIT0028] White CR, Smythies LE, Crossman DK, Palgunachari MN, Anantharamaiah GM, Datta G. 2012. Regulation of pattern recognition receptors by the apolipoprotein A-I mimetic peptide 4F. Arterioscler Thromb Vasc Biol. 32:2631–2639. 22982462 10.1161/ATVBAHA.112.300167 PMC4083685

[CIT0029] Willis BC, Liebler JM, Luby-Phelps K, Nicholson AG, Crandall ED, du Bois RM, Borok Z. 2005. Induction of epithelial-mesenchymal transition in alveolar epithelial cells by transforming growth factor-beta1: potential role in idiopathic pulmonary fibrosis. Am J Pathol. 166:1321–1332. 15855634 10.1016/s0002-9440(10)62351-6 PMC1606388

[CIT0030] Wilson MS, Wynn TA. 2009. Pulmonary fibrosis: pathogenesis, etiology and regulation. Mucosal Immunol. 2:103–121. 19129758 10.1038/mi.2008.85 PMC2675823

[CIT0031] Wynn TA, Barron L. 2010. Macrophages: master regulators of inflammation and fibrosis. Semin Liver Dis. 30:245–257. 20665377 10.1055/s-0030-1255354 PMC2924662

[CIT0032] Wynn TA, Ramalingam TR. 2012. Mechanisms of fibrosis: therapeutic translation for fibrotic disease. Nat Med. 18:1028–1040. 22772564 10.1038/nm.2807 PMC3405917

[CIT0033] You J, Wang J, Xie L, Zhu C, Xiong J. 2016. D-4F, an apolipoprotein A-I mimetic, inhibits TGF-β1 induced epithelial-mesenchymal transition in human alveolar epithelial cell. Exp Toxicol Pathol. 68:533–541. 27495007 10.1016/j.etp.2016.07.005

